# Coronary artery disease and the risk of life-threatening cardiac events after age 40 in long QT syndrome

**DOI:** 10.3389/fcvm.2024.1418428

**Published:** 2024-10-22

**Authors:** Alon Barsheshet, Ilan Goldenberg, Milica Bjelic, Kirill Buturlin, Aharon Erez, Gustavo Goldenberg, Anita Y. Chen, Bronislava Polonsky, Scott McNitt, Mehmet Aktas, Wojciech Zareba, Gregory Golovchiner

**Affiliations:** ^1^ Department of Cardiology, Rabin Medical Center, Petah-Tikva and the Faculty of Medicine, Tel Aviv University, Tel Aviv, Israel; ^2^ Clinical Cardiovascular Research Center, University of Rochester Medical Center, Rochester, NY, United States

**Keywords:** long QT syndrome, coronary artery disease, sudden cardiac death, ventricular arrhythmia, risk factors

## Abstract

**Background and aims:**

Long QT syndrome (LQTS) and coronary artery disease (CAD) are both associated with increased risk of ventricular tachyarrhythmia*.* However, there are limited data on the incremental risk conferred by CAD in adult patients with congenital LQTS. We aimed to investigate the risk associated with CAD and life threatening events (LTEs) in patients with LQTS after age 40 years.

**Methods:**

The risk of LTEs (comprising aborted cardiac arrest, sudden cardiac death, or appropriate defibrillator shock) from age 40 through 75 years was examined in 1,020 subjects from the Rochester LQTS registry, categorized to CAD (*n* = 137) or no-CAD (*n* = 883) subgroups.

**Results:**

Survival analysis showed that patients with CAD had a significantly higher cumulative event rate of LTEs from 40 to 75 years (35%) compared with those without CAD (7%; *p* < 0.001 for the overall difference during follow-up). Consistently, multivariate analysis showed that the presence of CAD was associated with a 2.5-fold (HR = 2.47; *p* = 0.02) increased risk of LTEs after age 40 years. Subgroup analyses showed that CAD vs. no CAD was associated with a pronounced >4-fold (*p* = 0.008) increased risk of LTEs among LQTS patients with a lower-range QTc (<500 ms). The increased risk of LTEs associated with CAD was not significantly different among the 3 main LQTS genotypes. Patient treatment was suboptimal, with only 63% on β-blockers and 44% on non-selective β-blockers.

**Conclusions:**

Our findings suggest that CAD is associated with a higher risk of LTEs in LQTS patients, with the risk being more pronounced in those with QTc <500 ms.

## Introduction

Congenital long QT syndrome (LQTS) is an inherited channelopathy characterized by the prolongation of action potential duration, which can lead to early after depolarizations and potentially life-threatening ventricular arrhythmias. Individuals carrying pathogenic or likely pathogenic variants of LQTS may experience palpitations, syncope, or sudden cardiac death. Previous studies have shown that the risk of life threatening cardiac events (LTEs) among patients with LQTS is influenced by age, gender, corrected QT interval (QTc), genotype, mutation location, environmental factors and therapy (1). The primary focus of these studies was on the initial four decades of life, leaving a scarcity of data regarding predictors of LTEs in individuals with LQTS beyond the age of 40 years (2). Possible factors that may affect the risk of life threatening arrhythmic events in LQTS patients especially after the fourth decade of life include comorbid conditions that accumulate during the years such as diabetes mellitus, hypertension, smoking, hyperlipidemia and coronary artery disease (CAD).

Beyond the age of 40 years, CAD becomes the primary cause of sudden cardiac death (SCD). Prior research has indicated that an abnormally prolonged QTc may also play a role as a causative factor in the pathophysiology of SCD in patients with CAD (3). Thus, in this study we sought to examine whether CAD contributes to the risk of life threatening arrhythmic events after the fourth decade of life beyond the risk associated with congenital LQTS during this time period.

## Methods

### Study population

The study population was drawn from the Rochester LQTS Registry and involved enrolled LQTS subjects who were followed up after the age of 40 years. Enrolled LQTS subjects had either genetically confirmed pathogenic or likely pathogenic LQTS variants or a QTc ≥450 ms in males or ≥470 ms in females. Patients with multiple LQTS-causing mutations (*n* = 32) and patients with LQTS genotype other than LQTS 1–3 genotype (*n* = 24) were excluded. The final study cohort comprised 1,020 patients.

### Data collection and follow-up

Clinical data were collected as previously described (2). Briefly, A comprehensive medical history was gathered upon enrollment, and continuous clinical data were collected using prospectively designed forms during each visit or medical contact at yearly intervals. The duration of the QT corrected for heart rate (QTc) by use of Bazett's formula was assessed from a 12-lead ECG obtained at enrollment and assessed again from ECGs recorded during follow up. The QTc that was chosen for the current study was determined by the maximum QTc duration recorded before the age of 40 years or by the first ECG recorded after the age of 40 in patients lacking prior ECG data.

Data on non-LQTS comorbidities were collected through baseline and follow-up questionnaires that are sent to enrolled subjects ≥40 years old at the yearly follow-up assessment. Patients were identified as having CAD if they had a history of acute coronary syndrome, coronary artery angioplasty, coronary artery bypass graft or had chest pain and were prescribed nitroglycerine. The presence of CAD was assessed as a time dependent variable (including the date when CAD diagnosis was made according to filled forms).

Follow-up data regarding β-blocker therapy included the initiation date and, if applicable, the discontinuation date. Information on other LQTS-related therapies including pacemaker or ICD implantation, and left cervical sympathetic denervation was also recorded prospectively.

Pathogenic or likely pathogenic LQTS variants were identified using standard genetic tests performed in academic molecular-genetic laboratories affiliated with the Rochester Long QT Syndrome Registry. Patients with variants of uncertain significance (VUS) were excluded from the study unless their QTc was ≥450 ms for males or ≥470 ms for females. Genetic testing included comprehensive LQTS gene panels or targeted mutation testing, utilizing methods such as Sanger sequencing, Next-Generation Sequencing (NGS), and occasionally Multiplex Ligation-dependent Probe Amplification (MLPA).

### Endpoints

The primary end point of the study was the occurrence of a first LTE, comprising aborted cardiac arrest (ACA) requiring external defibrillation as part of the resuscitation, LQTS-related SCD (abrupt in onset without evident cause if witnessed or death that was not explained by any other cause if it occurred in an unwitnessed setting) or an appropriate ICD shock between the ages of 40 and 75 years. Events that occurred during an acute myocardial infarction (MI) were excluded.

All participants provided informed consent, consenting to inclusion in the registry and participation in subsequent clinical studies.

### Statistical analysis

To explore the unadjusted relationship between LTE and time-dependent CAD, Simon and Makuch plot is displayed, which is an expansion of the Kaplan-Meier plot with respect to time-dependent covariates. Mantel-Byar test was used for comparison of survival data with a time-dependent covariate. To assess the independent association of clinical and genetic factors with the initial incidence of a LTE, multivariable Cox proportional hazards modeling was conducted with decade was born (before January 1970 and after January 1970) strata and robust standard errors were utilized to account for clustering of patients within family. Best stepwise variable selection method was applied selecting from the following list of potential covariates based on literature review and clinical judgement: Age, sex, QTc ≥500 ms, CAD, hypertension, diabetes mellitus, hyperlipidemia, mitral valve prolapse documented by echo, heart disease other than LQTS or CAD, asthma, autoimmune disorder, smoking, obstructive sleep apnea, menopause, cancer, high alcohol intake, syncope prior to age 40 years, time-dependent β-blocker use, and genotype mutations. The reduced model included all covariates at the <0.05 significant level, but forcing sex, history of syncope before age 40 years, time-dependent β-blocker use, and genotype mutations. Furthermore, specified subgroup analyses and test of interactions were investigated for LTE outcome.

All statistical analyses were two-sided, and significance was determined at a *p*-value of <0.05. The analyses were conducted using SAS software (version 9.4, SAS Institute, Cary, North Carolina).

## Results

### Clinical characteristics

The clinical characteristics of 1,020 study patients and the frequency of comorbidities before age 40 years and during follow up by CAD are presented in Table 1.

**Table 1:** Baseline and follow-up characteristics of the study population by coronary artery disease.

Clinical characteristics	CAD *n* = 137	No CAD *n* = 883	*P*-value
Female sex %	55	66	**0**.**022**
ECG parameters			
RR, ms	898 ± 193	900 ± 188	0.907
QT, ms	462 ± 66	456 ± 67	0.180
QTc, ms	491 ± 42	483 ± 49	**0**.**014**
QTc ≥500 ms %	36	30	0.166
Prior cardiac events before age 40 years			
Syncope%	30	37	0.118
Aborted cardiac arrest (ACA) %	5	6	0.833
Appropriate ICD shock %	1	2	0.710
Cardiac events%	31	39	0.077
ACA or appropriate ICD shock %	5	6	0.546
LQTS Genotype^a^			
LQT1 %	19	31	**<**.**001**
LQT2 %	26	32	
LQT3 %	9	8	
No mutation found (tested) %	11	8	
Unknown mutation status (not tested) %	34	20	
Comorbidities before age 40 years and during follow-up			
Sleep apnea %	9	7	0.498
Asthma %	23	15	**0**.**020**
Cancer %	12	8	**0**.**072**
Diabetes mellitus %	19	10	**0**.**003**
Hypertension %	62	33	**<**.**001**
Hyperlipidemia %	26	16	**0**.**004**
Stroke %	15	4	**<**.**001**
Smoking %	56	35	**<**.**001**
Therapies during follow-up after age 40 years			
β-blockers %	80	63	**<**.**001**
Selective β-blockers %	64	41	**<**.**001**
Non-selective β-blockers %	39	34	0.223
Pacemaker %	9	3	**0**.**002**
ICD %	33	19	**<**.**001**
LCTSD %	1	0	0.515
Cardiac events during follow-up after age 40 years			
Syncope %	29	14	**0**.**001**
ACA %	9	2	**<**.**001**
LQTS-related SCD %	0	0	1.000
Appropriate ICD shocks %	4	3	0.765
Cardiac event %	36	17	**<**.**001**
Life-threatening event %	12	5	**0**.**014**
All-cause mortality %	12	4	0.053

ACA, aborted cardiac arrest; ICD, implantable cardioverter-defibrillator; LCSD, left cervical sympathetic denervation; LQTS, long QT syndrome; SCD, sudden cardiac death; QTc, corrected QT.

^a^ A total of 800 (78%) subjects were genotyped. Of them, 710 (89%) subjects were identified as carriers of an LQTS 1-3 mutation.

*P*-values < 0.05 are highlighted in bold.

The 137 patients with CAD exhibited significant differences in baseline characteristics when compared with 883 patients with no-CAD. Patients with CAD had longer QTc duration, and displayed a higher frequency of comorbidities, including diabetes mellitus, hypertension, hyperlipidemia, smoking, history of stroke and asthma when compared to patients with no-CAD. Conversely, no-CAD patients displayed a greater proportion of females. Among patients with CAD, 43% had a history of MI, 63% underwent coronary angioplasty, and 25% underwent coronary artery bypass grafting.

### Genotype

Out of the total, 800 (78%) individuals underwent genotyping. Among them, 710 (89%) were found to carry an LQTS 1–3 mutation and were classified as LQT1, LQT2, or LQT3. The remaining 90 (11%) individuals who were genotyped but did not carry an LQTS mutation were classified as “no mutation found (tested)”. There were 220 (22%) LQTS patients that were not tested genetically and were thus categorized as unknown mutation status (not tested). Patients without CAD displayed a greater proportion of LQT1 and LQT2 related mutations compared with patients with CAD.

### Clinical course of LQTS patients with CAD vs. no CAD after age 40 years

During a mean (±SD) follow up of 21.4 (±11.7) years, 97 patients experienced any cardiac event, and 60 patients experienced an LTE after age 40. Among the 60 patients who experienced an LTE, 29 had an ACA, 4 experienced SCD related to LQTS, and 33 received appropriate shocks from an ICD. Patients with CAD more frequently received β-blockers (mainly selective β-blockers), pacemakers and ICDs when compared to no-CAD patients. The proportion of patients who experienced syncope, ACA, any cardiac event, LTEs or death was significantly higher in the CAD vs. no CAD group (Table 1).

**Figure 1:**
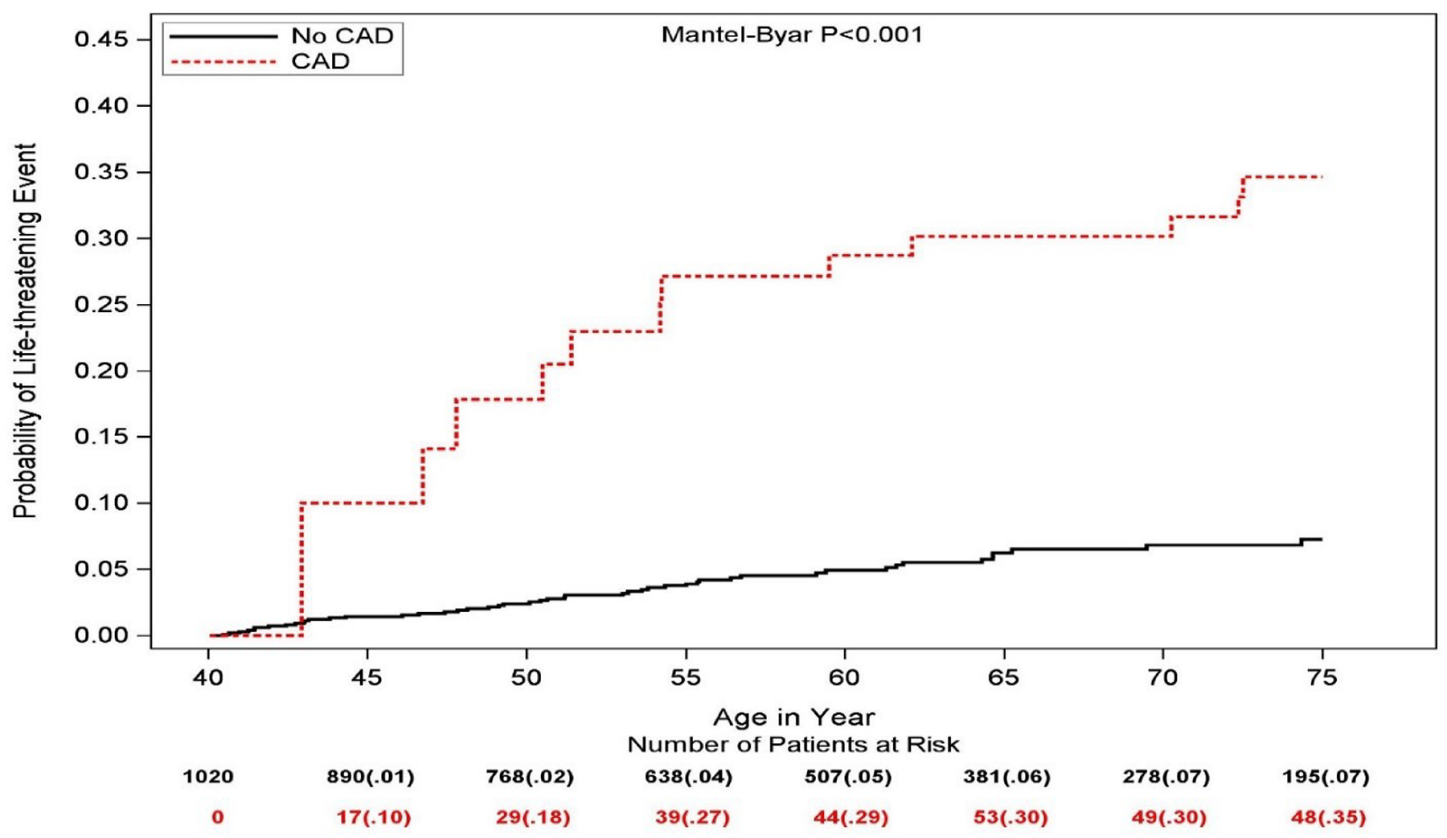
Simon and Makuch estimates for life threatening cardiac events by time-dependent coronary artery disease (values in parentheses are probability estimates).

Figure 1 presents the Simon and Makuch estimates for LTEs by time-dependent CAD. The cumulative probability of LTE at age 75 was significantly higher in CAD patients (35%) than among no-CAD patients (7%, Mantle-Byar *p* < 0.001).

Multivariate analysis (Table 2) consistently showed that the presence of CAD was a powerful predictor of LTEs in LQTS patients after age 40 years and was independently associated with a 2.5-fold increased risk of LTE (*p* = 0.02). A more prolonged QTc (≥500 ms) was also identified to be a powerful independent predictor of LTE (HR = 3.54; *p* < 0.001), whereas other known LQTS related known risk factors, including sex, prior syncope, and genotype were not associated with a statistically significant increased risk after adjustment for the presence of CAD (Table 2).

**Table 2:** Multivariate analysis: risk factors for life threatening cardiac events among all LQTS patients^a^.

Risk factor	HR	95% CI HR	*P*-value
QTc ≥500 ms	3.54	2.09–6.00	<.0001
Coronary artery disease	2.47	1.15–5.30	0.020
Women vs. men	1.17	0.67–2.05	0.573
Syncope prior to age 40 year	1.12	0.64–1.96	0.697
LQTS genotype^b^			
LQT2 vs. LQT1	1.75	0.91–3.34	0.092
LQT3 vs. LQT1	0.90	0.30–2.71	0.858
No mutation found (tested) vs. LQT1	1.23	0.42–3.54	0.707
Unknown mutations (not tested) vs. LQT1	1.12	0.51–2.42	0.782

^a^ Life threatening cardiac events indicate aborted cardiac arrest, sudden cardiac death, or ICD shock. Models were further adjusted for time-dependent β-blockers therapy and stratified for decade of birth.

^b^ The global *p*-value = 0.420 for testing the association between LQTS genotype and ACA/SCD/ICD shock.

Other CAD risk factors such as hypertension, diabetes mellitus, and hyperlipidemia were not associated with a statistically significant increased risk of LTEs and therefore were not included in the final LTE model (Table 2).

### Subgroup analysis

**Figure 2:**
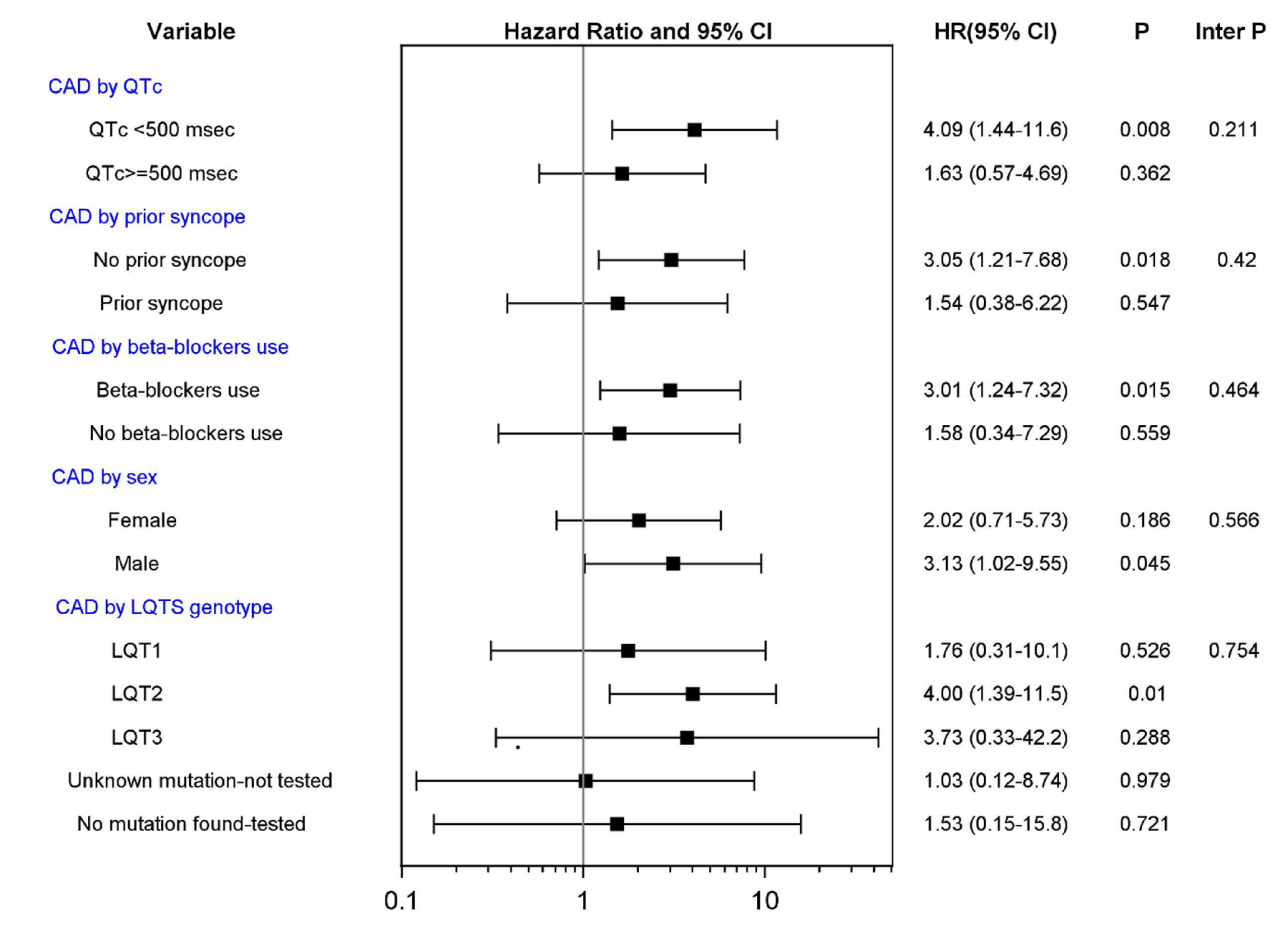
CAD vs. no CAD and risk of life threatening cardiac events in subgroups of patients. CAD, coronary artery disease; LQTS, long QT syndrome. Tests of interactions were performed using a single interaction term, except for the model investigating CAD by LQTS genotype using 4 interaction terms.

**Figure 3:**
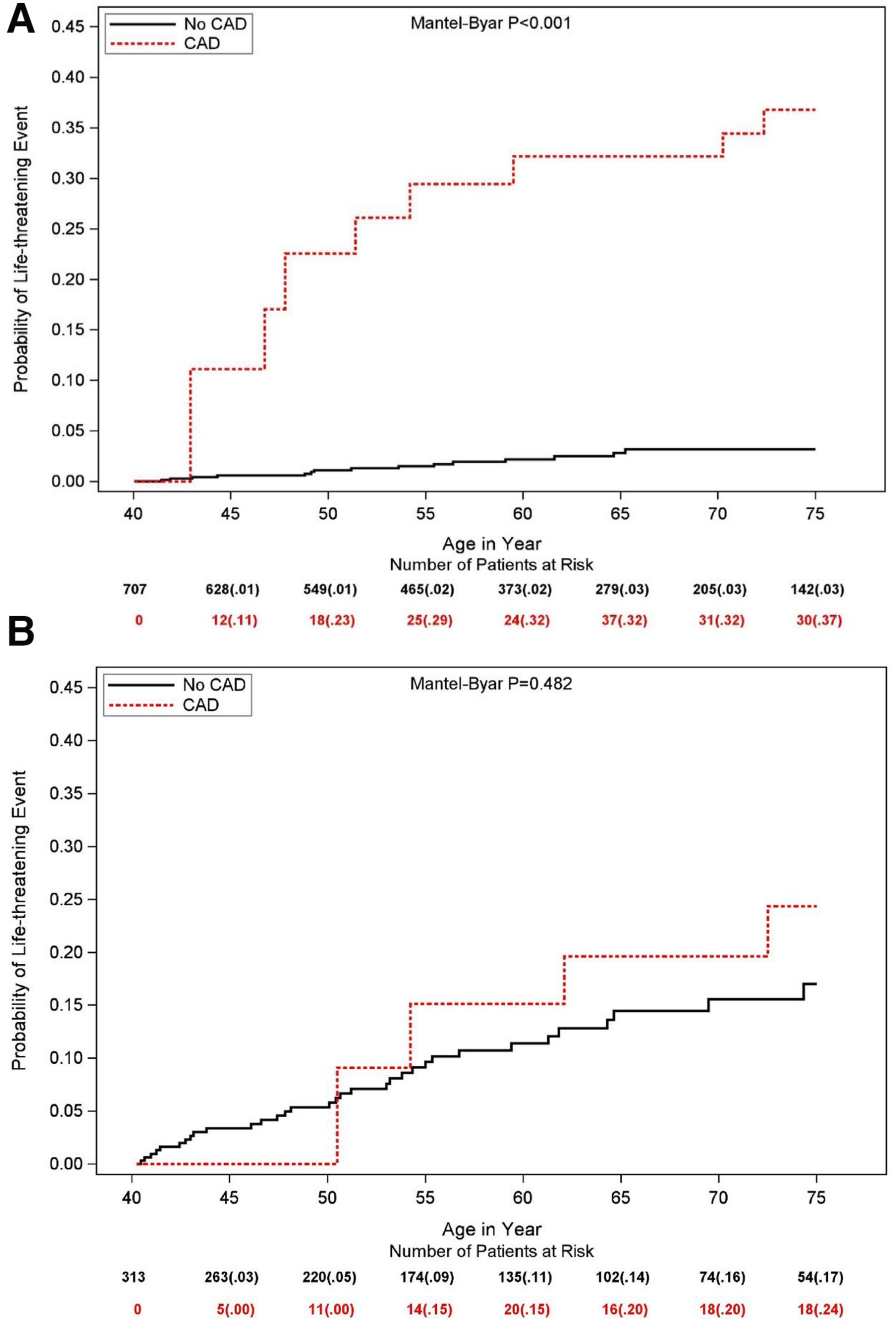
Simon and Makuch estimates for life threatening cardiac events by time-dependent coronary artery disease in patients with **(A)** QTc <500 ms and **(B)** QTc ≥500 ms. Values in parentheses are probability estimates.

Subgroup analysis (Figure 2) did not show a statistically significant difference in the correlation between CAD and LTE across risk subsets of LQTS patients, encompassing QTc duration, history of syncope prior to age 40 years, β-blocker utilization, gender, and LQTS genotype (all *p*-values for risk subset-by-CAD interaction >0.10). Nevertheless, the increased risk of LTEs associated with CAD was pronounced (>4-fold risk increase) in lower risk LQTS patients (with QTc <500 ms) and among those with the LQT2 genotype (Figure 2). Thus, among patients with QTc <500 ms, the cumulative risk of LTEs from age 40 to 75 was 37% in the CAD vs. only 3% in the no CAD subgroup (*p* < 0.001 for the overall difference during follow-up (Figure 3A). In contrast, among patients with QTC ≥500 ms, there was no statistically significant difference in the cumulative risk of LTE between the CAD (24%) and no-CAD (17%) subgroups (*p* = 0.482, Figure 3B).

In an exploratory analysis, we reviewed data on unaffected family members of LQTS patients who were followed after age 40 from the LQTS Registry. These individuals did not have LQTS (no confirmed LQTS-causing genetic mutation and no QTc ≥450 ms in males or ≥470 ms in females). Among the 599 subjects, 55 had CAD and 544 did not. Over a mean follow-up period of 22 ± 12 years, there were only 4 LTE episodes based on the earlier definition. Two patients with CAD and two without CAD experienced appropriate ICD shocks. This very low LTE rate among LQTS-negative family members highlights that the original cohort of LQTS patients included true LQTS-related life-threatening events, rather than ACS-related or scar-related LTEs.

## Discussion

Our study provides several important implications for risk stratification and management of patients with long QT syndrome after age 40 years. We have shown that from age 40 to 75 years the presence and/or development of CAD is associated with a pronounced incremental risk for LTE compared to the risk associated with LQTS in this age-group without CAD. These findings were independent of known LQTS risk factors and were consistent in all risk subsets analyzed. Nevertheless, the incremental risk associated with CAD appears to be very pronounced (≥4-fold risk increase) in lower risk LQTS patients (with QTc <500 ms). These findings suggest that more careful follow-up and monitoring for arrhythmic events is required in adult LQTS patients who develop CAD.

### Incremental effect of CAD in LTE in adult patients with LQTS

Most studies evaluating the clinical course and prognosis of subjects with LQTS have focused on the first 4 decades of life. Although it has been suggested that carriers of this congenital disorder have a low risk of ventricular arrhythmias or SCD after the age of 40 years, research conducted within the international LQTS cohort demonstrated that individuals carrying an LQTS mutation faced a fourfold higher risk of LTEs between the ages of 40 and 60 compared with genotype-negative subjects in the same age group (2).

There is lack of data on the effect of cardiovascular comorbidities on the risk of LTEs among LQTS patients after the age of 40 years. A previous study analyzing data from the International Long QT Syndrome Registry has suggested that CAD may augment the risk of cardiac events in LQTS patients at the age range of 41–80 years (4). In this study we included 641 LQTS patients (of whom 87 patients had CAD); CAD vs. no CAD was associated with a 2-fold increased risk of cardiac events dominated by syncope. In this study, however, the risk of LTEs was not assessed due to a smaller sample and a lower event rate. The present study including 1,020 LQTS patients found that CAD was associated with a a statistically significant 2.5-fold increase in the risk of LTEs, thus supporting previous findings and extending the results for the robust endpoint of LTEs.

Diabetes mellitus may also affect ventricular repolarization (5, 6); Ouelett G. et al, showed that the development of diabetes mellitus in adult patients with LQTS was not associated with an increased risk of any cardiac events (dominated mainly by syncope events) (7). In our study, focusing on the endpoint of LTEs, we found similar results, that diabetes mellitus was not associated with increased risk of LTEs among LQTS patients. Furthermore, additional established CVD risk factors, including smoking and hypertension were not shown to confer increased risk for LTE in our LQTS population after further adjustment for the presence of CAD.

In the current study, 6% of the patients experienced an LTE, with a higher incidence observed in those with CAD (12%) compared to those without CAD (5%). Thus, it appears that the rates of LTEs are somewhat higher than those reported in studies with follow-up before age 40 (8, 9).

Additionally, the use of β-blockers and left cardiac sympathetic denervation (LCSD) after age 40 and during follow-up was less frequent than in studies involving patients under 40 (8, 10, 11). However, direct comparison between these studies and the current one is challenging due to differences in patient age, characteristics, QT interval lengths, follow-up duration, and endpoint definitions.

It should be noted that the population in this study was not optimally treated with β-blockers: only 63% of patients received β-blockers, and just 44% were treated with non-selective β-blockers. Patients with CAD more frequently received β-blockers (mainly selective beta blockers) compared to no-CAD patients.

### Possible mechanisms linking CAD risk in LQTS

There are several pathophysiological substrates that contribute to the development of LTEs in patients with CAD including arrhythmias initiated by transient ischemia, arrhythmias occurring during the acute and convalescent phases of MI, after scar formation and in the setting of ischemic cardiomyopathy (3). Previous studies have suggested that abnormally prolonged ventricular repolarization may be a causative factor in the pathophysiology of SCD in patients with CAD. Chugh et al. have found that an abnormal QT_C_ prolongation (>470 ms) was associated with a 5-fold increased risk of SCD in a population based case-control study of patients with CAD (12). In addition, clinical and experimental evidence show that ischemia/reperfusion injury is associated with prolongation of repolarization in the affected area that may contribute to the generation of triggered activity leading to polymorphic ventricular tachycardia (13). It is possible that there is a mechanistic link between QT prolongation associated with ischemia/reperfusion injury and the abnormal regulation of potassium, sodium, and calcium ions flux across myocellular membranes as appears in the congenital long QT syndrome, thus facilitating the generation of triggered activity leading to ventricular arrhythmias.

### Effect of QTc duration and genotype on CAD-related risk

In the current study we found that the presence or development of CAD was associated with a statistically significant pronounced increased risk of subsequent LTEs among patients with QTc <500 ms (HR = 4.09, *p* = 0.008). The mechanism underlying the observed pronounced increase in the risk associated with CAD in lower risk LQTS patients (i.e., with a shorter QTc duration) is unclear. It is possible that patients with a very prolonged QTc (≥500 ms) have already a very high risk of LTE, regardless of the presence of CAD, while the effect of ischemia/injury on repolarization dynamics is more pronounced in LQTS patients who have a baseline lower risk for arrhythmic events. Adult LQTS patients with QTC <500ms are considered to have low risk for cardiac events and therefore may be followed, monitored and treated (with β-blockers) less intensively than patients with QTc ≥500 ms, but when they develop CAD their risk for cardiac events increases dramatically.

### Limitations

The Rochester Long QT Syndrome Registry is continually collecting follow up data on non-LQTS comorbidities including CAD for enrolled subjects older than 40 years since January 2004. For the diagnosis of CAD we relied on data from prespecified medical questionnaire. We did not collect data on the severity of CAD per coronary angiogram, data on multiple PCI procedures, residual inducible ischemia or type of MI and thus were not able to perform subgroup analyses according to CAD severity. There were 220 (22%) LQTS patients that were not tested genetically and were thus categorized as unknown mutation status (not tested). We did not gather data on left ventricular ejection fraction. While we did not have complete data on all episodes of ventricular arrhythmias, among the 60 patients who experienced LTEs, 29 episodes of VF and 20 episodes of Torsades de Pointes were recorded, suggesting that most events were related to LQTS rather than monomorphic ventricular tachycardia (VT). Nevertheless, we cannot rule out that the larger risk of LTE among CAD patients with QTc <500 ms might be related to a higher incidence of scar-related monomorphic VT. There is a high rate of LTEs among LQTS patients with CAD in the present study compared to other studies that could be related to age-related factors and follow-up duration. However, it is still possible that non-LQTS-related LTEs were included, despite our careful review of this endpoint. We also did not collect data on whether an electrophysiological study was conducted following syncopal events.

### Clinical implications and future directions

The present study is among the first to assess the relationship between CAD and life-threatening cardiac arrhythmic events among LQTS patients after the age of 40 years.

In this study we demonstrate that CAD contributes to the risk of life threatening cardiac events beyond the risk conferred by congenital LQTS. CAD was associated with a 2.5 fold increased risk of LTEs after age 40. We suggest that LQTS patients who develop CAD are at increased risk for LTEs and should be followed, monitored and treated more intensively (focusing on dose optimization of nonselective β-blockers). Further research is needed to understand determinants and mechanisms underlying the increased risk of cardiac arrhythmias in these patients.

## Data Availability

The data analyzed in this study is subject to the following licenses/restrictions: no public access. Requests to access these datasets should be directed to Barshesheta@gmail.com.
